# Prevalence of Methylenetetrahydrofolate Reductase C677T Polymorphism in women with Polycystic Ovary Syndrome in southeast of Iran

**Published:** 2015

**Authors:** A Naghavi, H Mozdarani, M Garshasbi, M Yaghmaei

**Affiliations:** * Department of Medical Genetics, Faculty of Medical Sciences, Tarbiat Modares University, Tehran, Iran,; ** Department of Obstetrics and Gynecology, Faculty of Medical Sciences, Shahid Beheshti University, Tehran, Iran

**Keywords:** Methylenetetrahydrofolate reductase, C677T polymorphism, Polycystic Ovary Syndrome

## Abstract

**Background:** One of the notable enzymes in the metabolism of folate is Methylenetetrahydrofolate reductase enzyme, this enzyme is necessary for some biological mechanisms. Mutations in the MTHFR gene could reduce the enzyme activity.

**Aim:** The objective of this research was to assess the prevalence of the very general polymorphism, C677T, in females with polycystic ovary syndrome in the southeastern of Iran.

**Methods:** This research was a case–control research and was conducted on 112 PCOS women and 196 healthy controls. Single type nucleotide polymorphisms (SNP) were genotyped by employing the polymerase chain reaction–restriction fragment length polymorphism (PCR–RFLP).

**Results:** It noticed that in C677T, the pervasiveness of C/ C, C/ T, and T/ T genotypes was 54.5%, 34%, and 11.5%, respectively. The repetition of TT genotype was notably higher in PCOS women contrasted to controls.

**Conclusions:** the appearance of 677T allele could be a danger agent for PCOS susceptibility in the southeast of Iran.

## Introduction

One of the very frequent endocrine disorders is polycystic ovary syndrome (PCOS) whidisordersch affecting 5% to 10% of the females in generative age based on the Rotterdam criteria. PCOS is identified by a couple of the coming next abnormalities: hyperandrogenism, amenorrhea or oligomenorrhea and polycystic ovaries. PCOS is connected with overweight, class 2 diabetes, metabolic syndrome, the disease of heart, and endometrial cancer risk [**1**,**2**]. The exact etiology and pathophysiology of PCOS have not been completely identified. However, there is strong evidence for a genetic base for it [**3**]. Several types of research were conducted on the connection between PCOS and genetic factors. There are several reports about the connection between MTHFR polymorphisms and PCOS in which their outcomes are variable [**4**-**7**].

MTHFR is an essential regulative enzyme in folate metabolism that is required for some of the biological systems. This enzyme activates the decrease about 5, 10-methylenetetrahydrofo-late into 5-methyltetrahydrofolate. This MTHFR gene, placed in the small arm of chromosome 1 (1p36.3), is included of 11 exons. Two normal allelic variants within MTHFR gene, are C677T (A222 V, rs1801133) and also A1298C (E429A, rs1801131). Both these polymorphisms could decrease the enzyme activeness. The C677T (Ala222Val) polymorphism in exon four generates an amino acid of the Alanine kind for replacement of Valine in the catalytic field of the N-terminal . This differing generates a thermolabile protein with the enzymatic activeness which is reduced by 70% in the homozygote status (TT genotype) and 35% in the heterozygote status (CT genotype) [**8**,**9**]. The A1298C (Glu429Ala) replacement is in the regulative region of the MTHFR enzyme and may change the enzyme behavior[**10**-**11**]. 

In the current investigation, the pervasiveness of MTHFR C677T polymorphism in PCOS patients in the Southeast of Iran was analyzed and correlated with controls of healthiness. 

## Materials and methods 

**Subjects characteristics**

In current case-control research, 308 women including 112 patients that have polycystic ovarian syndrome and 196 healthy individuals from Obstetrics and also Gynecology Center from Emam-Ali Hospice in Zahedan, Iran, were recruited. This survey was supported by the Ethics Board of the University of Tarbiat Modares and the consensus forms were gathered for all the individuals. PCOS was deﬁned based on the Rotterdam models. The checking subject collection involved 196 unrelated age-matched normal women. All PCOS subjects and normal controls were nonsmokers. Patients who suffered from diabetes mellitus, thyroid disorder, hyperprolactinemia, hypertension, Cushing’s syndrome, premature ovarian failure, acromegaly, virilising, adrenal, or ovarian tumors were dropped from the research. 

**Genotype analysis**

Blood specimens were gathered inside cylinders including an anticoagulant (disodium-EDTA), furthermore settled in -20-degree celsius till DNA extraction. And genomic DNA was plucked by the standard kite. The C677T variant of MTHFR was magnified by using a progressive primer 5-GGTCAGAAGCATATCAGTCATGAG-3 and a backward primer 5 – CTGGGAAGAACTCAGCGAACTCAG - 3 by the PCR. PCR situations were denaturation at 95-degree celsius and as long as 300 seconds, 30 cycles at 95-degree celsius and as long as 30 seconds, 62-degree celsius and as long as 30 seconds, and 72-degree celsius and for 30 seconds followed by continuation at 72-degree celsius and as long as 7 min. 

The PCR outcomes (494bp) were digested with Hinf I in 37°C night long and electrophoresed on a 2% agarose gel. The two fragments, 100bp, and 394bp showed the homozygosity for the C allele, the three pieces, 100bp and 165bp and 229bp showed homozygosity for the T allele and the four parts, 100bp, 165bp, 229bp and 394bp showed heterozygosity in the T and C alleles. Genotypes displayed as CC in exchange for homozygous common, CT in exchange for heterozygous, furthermore TT in exchange for the homozygous mutant. 

**Analytical examination:** all analytical examination were conducted with SPSS V-20. Analytical and clinical differences among the two collections were tested by independent learner's t-test, Mann Witney u test, or Fisher exact examination when proper. Allele frequencies were determined by the gene numerate process. The odds rate (OR) and 95% intervals of certainty were similarly calculated. The χ2 examination was applied to the difference of genotype dissemination from Hardy-Weinberg equality. 

## Results

308 women including 112 (36.4%) subjects with polycystic ovarian syndrome and 196 (63.6%) healthy individuals, were recruited. The demographic characteristics of PCOS females and controls were presented in **Table 1**. The PCOS subjects and checking subjects did not differ notably with regard to lifetime and race. The alleles and genotypes prevalence of MTHFR C677T polymorphism are presented in **Table 2**. No variation from equilibrium of Hardy-Weinberg was recognized in the PCOS women and control group.

**Table 1:** Demographic features of PCOS females and checking subjects

	PCOS women (n=62)	checking subjects (n=196)	P‏
Age (Years)	25.4 ± 5.2	26.4 ± 5.6	0.2
Race, n (%)			
Fars	43(69)	142(72)	
Balouch	19(31)	54(28)	

**Table 2:** Alleles and genotypes frequency of MTHFR C677T polymorphism

	PCOS women (n=112)	checking subjects (n=196)	P‏	OR (95% CI)	P‏ *	OR (95% CI)*
MTHR (C677T)						
CC, n (%)	61(54.5)	136(69.4)		1		
CT, n (%)	38(34)	51(26)	0.055	1.7 (1–2.8)	0.06	1.7 (1–2.8)
TT, n (%)	13(11.5)	9(4.6)	0.01	1.8 (1.1–2.8)	0.02	1.7(1.1-2.7)
CT+TT, n (%)	51(45.5)	60(30.6)	0.009	2(1.2-3.1)	0.01	1.9(1.2-3.1)
Allele						
C, n (%)	160(71)	336(82)				
T, n (%)	64(29)	56(18)	<0.0001	2.4(1.6-3.6)		
*Adjusted for age and ethnicity						

Although the frequency of the CT genotype of MTHFR C677T polymorphism was not statistically varied among PCOS and control females before and after adjusting age and ethnicity, the frequency of TT genotype was notably greater in PCOS women contrasted to controls before and after setting lifetime and ethnicity and risk of PCOS was 1.7 fold greater in women with TT genotype after setting lifetime and ethnicity [OR, 1.7 (95% CI 1.1 to 2.7); P = 0.02]. In addition, the frequency of 677T allele was 29% and 18% respectively, in PCOS women and control groups, which was numerically altered (p<0.0001). Moreover, the all over prevalence of individuals with 677T allele (CT and TT genotypes) were notably greater in the PCOS cases contrasted to checking subjects (45.5 vs. 30.6%,) and the presence of T allele could be a risk factor for PCOS susceptibility (OR, 1.9 [95% CI, 1.2 to 3.1]; P = 0.01).

## Discussion

Different gene polymorphisms have been recognized related with increased susceptibility for PCOS but due to the ethnic and conditional variations in populations, conflicting results have been obtained. Some writers have hypothesized the relationship of MTHFR variants, such as C677T and A1298C polymorphisms, with PCOS [**12**-**14**]. The current investigation tried to estimate the connection of MTHFR C677T polymorphism with PCOS in the southeast of Iran. 

308 women including 112 patients with polycystic ovarian syndrome and 196 healthy individuals were recruited. The results of the current research revealed that patients with PCOS, had notably higher numbers of TT in opposition with the healthy individuals. In a comparable observation by Qi Q et al., a significant connection was revealed between MTHFR gene C677T polymorphism with PCOS, for which CT and TT genotypes can raise the risk of PCOS [**15**]. Another study indicated that the 677T allele raises the PCOS susceptiveness, and that connection seemed to be extra strong in Europeans compared to Asians [**16**]. In other societies, varying results were found. Our findings were in contrast with the results of Kazeruni et al. on the Iranian population. Based on their results, the prevalence of C677T polymorphism was not found to be notably altered between the study groups [**17**]. In a case-control research, Jain et al. showed that no homozygous mutation (TT) was detected in the research population. Even if not statistically meaningful, there was a somewhat greater currency of heterozygous (CT) genotype in females with PCOS [**6**]. In a study by Karadeniz et al. [**15**], the MTHFR 677 CC genotypes had notably greater proportions in the control community contrasted to the PCOS cases in the Turkish population [**18**]. In another study by Choe et al. among the Korean population, the C677T polymorphism of MTHFR gene was not associated with PCOS. The conflicting results regarding the connections within MTHFR C677T polymorphisms and risks for PCOS may be as a result of differences in racial and environmental factors.
